# Vaping Cessation Methods Used by Young Adults

**DOI:** 10.1001/jamanetworkopen.2025.12803

**Published:** 2025-05-29

**Authors:** Brian S. Williams, Michael C. Fiore, Andrew Hyland, Wendy S. Slutske

**Affiliations:** 1 Center for Tobacco Research and Intervention, University of Wisconsin School of Medicine and Public Health, Madison; 2 Division of Hospital Medicine, Department of Medicine, University of Wisconsin School of Medicine and Public Health, Madison; 3 Division of Hospital Medicine and Complex Care, Department of Pediatrics, University of Wisconsin School of Medicine and Public Health, Madison; 4 Department of Health Behavior, Roswell Park Comprehensive Cancer Center, Buffalo, New York; 5 Department of Family Medicine and Community Health, University of Wisconsin School of Medicine and Public Health, Madison

## Abstract

This cross-sectional study describes past-year electronic nicotine product quit methods used by young adults.

## Introduction

Electronic nicotine products (ENPs) are the most popular commercial tobacco product used by young adults (YAs) aged 18 to 24 years.^1^ Their ENP use is concerning, as this population is at risk for long-term nicotine dependence and potential adverse health consequences.^2^ Evidence on effective ENP cessation treatment is lacking and little is known about how YAs are attempting to quit. Identifying ENP quit methods used by YAs can inform treatment development. This study aimed to describe past 12-month ENP quit methods used by YAs who participated in the Population Assessment of Tobacco and Health (PATH) study, a nationally representative cohort study.^3^

## Methods

We analyzed wave 7 data from the PATH study collected between January 2022 and April 2023.^3^ Participants were aged 18 to 24 years, reported ENP use in the past 12 months, reported an ENP quit attempt or successful ENP quit within the past 12 months, and had a wave 7 weight. Weights were used to provide US population estimates. The University of Wisconsin School of Medicine and Public Health Institutional Review Board deemed this cross-sectional study exempt from review and waived informed consent, as it was secondary analysis of an existing dataset. We followed the STROBE reporting guideline.

Cessation methods assessed included (1) social support (relying on friends and family); (2) behavioral support; (3) an application on a tablet or smartphone; (4) nicotine product substitution; (5) nicotine replacement therapy (NRT); and (6) prescription medications. Wave 7 cross-sectional weights were used along with replicate weights and balanced repeated replication for variance estimation.^3^ Details about participant demographics and the statistical analyses are provided in the eMethods in Supplement 1. Results are presented as unweighted numbers and weighted percentages. Analyses were performed using StataNow, version 18.5 (StataCorp).

## Results

Wave 7 of the PATH study included 10 310 YAs. In all, 3024 participants (30.6%) reported past 12-month ENP use and 855 (29.1%) (52.3% female; mean [SD] age, 20.9 [1.9] years) had quit or attempted to quit ENP use in the past 12 months (Table 1). Of 855 participants, 20.0% reported no ENP use in the past 30 days, suggesting that 80.0% of the quit attempts were unsuccessful. Social support was the most common quit method reported (29.8%), followed by tobacco product substitution (11.0%), with nicotine pouches (4.8%) and cigarettes (3.6%) being the most substituted products. Of individuals who used cigarettes as a substitution method to quit ENPs, all had a history of smoking. Less common quit methods were behavioral support (9.6%), smartphone or tablet application use (8.9%), and NRT (5.0%), with gum (2.4%) and patches (2.0%) being the most common NRT products used. The least frequently used quit method was prescription medications (1.7%). (Table 2).

**Table 1.** Demographic Characteristics of YAs Who Reported Quitting or Attempting to Quit ENPs in the Past 12 Months

Characteristic	Participants		
	Unweighted, No.	Weighted, No.	Weighted % (95% CI)
Total YA sample	10 310	29 419 729	NA
ENP users, any (past 12 mo)	3024	8 993 229	30.6 (29.3-31.9)
Dual use (ENPs and cigarettes, past 12 mo)^a^	1280	3 914 170	43.5 (41.4-45.7)
ENP quitter or attempter^a^	855	2 616 241	29.1 (27.5-30.7)
Sex			
Female	450	1 367 889	52.3 (48.6-56.0)
Male	405	1 248 352	47.7 (44.0-51.5)
Race^b^			
Asian race alone	22	100 582	3.8 (2.5-5.9)
Black alone	71	183 787	7.0 (5.4-9.1)
White alone	628	1 991 799	76.1 (73.3-78.7)
Other^c^	134	340 074	13.0 (11.0-15.2)
Ethnicity			
Hispanic	206	467 626	17.9 (15.7-20.3)
Non-Hispanic	649	2 148 615	82.1 (79.7-84.3)
Sexual orientation			
Gay	34	100 402	3.8 (2.5-5.7)
Straight	594	1 813 605	69.3 (65.7-72.7)
Bisexual	164	517 176	19.8 (16.9-23.0)
Something else	28	84 876	3.2 (2.2-4.8)
Not sure	35	100 183	3.8 (2.6-5.7)
Health insurance			
Yes	723	2 248 811	86.0 (83.1-88.4)
No	132	367 430	14.0 (11.6-16.9)

Abbreviations: ENP, electronic nicotine products; NA, not applicable; YA, young adults.

^a^ Weighted percentage based on the denominator of past 12-month ENP use.

^b^ Data on race and ethnicity along with sexual orientation were assessed to assist in developing population-based weights and specific subgroup analyses on tobacco use. Specific race response options and groupings are provided in the eMethods in Supplement 1.

^c^ Includes Guamanian or Chamorro, Native Hawaiian, Samoan, Other Pacific Islander, or multiracial.

**Table 2.** Electronic Nicotine Product (ENP) Quit Methods Used by Young Adults Who Reported Quitting or Attempting to Quit in the Past 12 Months

Quit method^a^	Participants, No.		Participants, % (95% CI)	
	Unweighted	Weighted	Weighted overall sample	Weighted within group
Social support				
Yes	252	779 186	29.8 (26.0-33.9)	NA
No	600	1 829 036	69.9 (65.7-73.8)	
Behavioral support^b^				
Yes	77	252 358	9.6 (7.5-12.3)	NA
No	776	2 360 824	90.2 (87.6-92.4)	
Smart phone or tablet application				
Yes	74	231 763	8.9 (7.0-11.1)	NA
No	779	2 381 419	91.0 (88.7-92.2)	
Product substitution^c^^,^^d^				
Yes	88	287 668	11.0 (8.9-13.5)	NA
Nicotine pouches	37	125 116	4.8 (3.4-6.7)	43.5 (32.3-55.5)
Cigarettes	30	94 083	3.6 (2.4-5.4)	32.7 (22.1-45.5)
Other oral tobacco	15	50 954	1.9 (1.2-3.2)	17.7 (10.5-28.3)
None	763	2 318 876	88.6 (86.0-90.8)	NA
NRT use^d^				
Yes	40	130 940	5.0 (3.6-6.9)	
Gum	19	61 728	2.4 (1.5-3.7)	47.1 (32.2-62.6)
Patch	16	51 772	2.0 (1.1-3.5)	39.5 (23.5-58.1)
Lozenge	7	22 233	0.8 (0.4-1.9)	17.0 (7.8-33.1)
Inhaler or spray^e^	NR	NR	NR	NR
None of the above	6	25 675	1.0 (0.5-2.1)	19.6 (9.3-36.8)
None	814	2 483 368	94.9 (93.1-96.3)	NA
Prescription medications^d^				
Yes	12	43 169	1.7 (0.9-3.1)	NA
Varenicline^f^	NR	NR	NR	NR
Bupropion	8	29 907	1.1 (0.5-2.6)	69.3 (37.4-89.5)
None of the above	3	10 954	0.4 (0.1-1.2)	25.4 (7.2-60.0)
No	842	2 570 926	98.3 (96.8-99.1)	NA

Abbreviations: NA, not applicable; NR, not reported; NRT, nicotine replacement therapy.

^a^ Results not provided for “don’t know” or “refused to answer.”

^b^ Behavioral support included counseling, telephone help line or quit line, books, pamphlets, videos, quit tobacco clinic, class, support group, internet or web-based program.

^c^ Results not displayed for traditional cigars, cigarillos, filtered cigars, pipe, hookah, snus, smokeless tobacco, or heated tobacco products due to low rates of reporting (<0.4% for each product).

^d^ Respondents could report using more than one product.

^e^ Nicotine inhaler and nicotine spray did not meet minimum threshold for reporting.

^f^ Varenicline did not meet the minimum threshold for reporting.

## Discussion

Our analysis of nationally representative data from the PATH study suggests that nearly one-third of US YAs (about 9 million) used ENPs in 2022, and nearly one-third of YAs who used ENP over the past 12 months (over 2.5 million) tried to quit. A small percentage of YAs used evidence-based smoking cessation interventions in their ENP quit attempts. Support from friends and family was the most popular method reported; less than 7% reported using NRT or prescription cessation medications. The small percentage of YAs using cessation medications was unlikely to be explained by lack of insurance given that more than 80% reported that they had coverage. Nicotine product substitution was reported by over 10% of the sample with nicotine pouches and combusted cigarettes most substituted. More research is needed to assess if pouch substitution is an effective harm-reduction strategy. There have been recent promising results supporting the use of a texting program^4^ and varenicline^5^ in ENP cessation, but our results suggest low uptake of these methods. More frequent use of social support by young adults suggests this may be an important component of future cessation treatment programs. Limitations include reliance on self-reported data and the potential for recall bias. Given the popularity of ENPs among YAs, there is a need for both evidence-based cessation treatments and improved implementation of effective treatments to help reduce ENP use.

## Notes

Supplement 1.
eMethods.

Supplement 2.
Data Sharing Statement
