# Supplementary material for: Vaping Cessation Methods Used by Young Adults
Source: JAMA Netw Open. 2025 May 29;8(5):e2512803. doi: 10.1001/jamanetworkopen.2025.12803 (PMC12123465; doi:10.1001/jamanetworkopen.2025.12803)
Supplement: Supplement 1. — eMethods. [file jamanetwopen-e2512803-s001.pdf]

Supplemental Online Content

Williams BS, Fiore MC, Hyland A, Slutske WS. Vaping Cessation Methods Used by Young Adults. *JAMA Netw Open.* 2025;8(5):e2512803.
doi:10.1001/jamanetworkopen.2025.12803

eMethods.

This supplemental material has been provided by the authors to give readers additional information about their work.

© 2025 Williams BS et al. *JAMA Network Open*.

eMethods

The PATH Study is a collaboration between the National Institutes of Health (NIH), National Institute on Drug Abuse (NIDA), Food and Drug Administration (FDA), and Center for Tobacco Products (CTP). The longitudinal study is administered by Westat®, who collected the survey information and provided weight calculations. The PATH Study data set is stored at the National Addiction & HIV Data Archive Program (NAHDAP) with more information found at: (https://www.icpsr.umich.edu/web/NAHDAP/series/606). Our study used the restricted use files from Wave 7. The PATH Study was reviewed and approved by the Westat IRB. Our study was reviewed by the University of Wisconsin School of Medicine and Public Health IRB and received an 'exempt' status. Informed consent for study participation was obtained by the PATH Study field interviewer prior to study enrollment. For Wave 7, data was collected via in-person, telephone, and web-based interviews.

Study participants self-reported basic demographic information during the survey response process. Race was assessed via the questions "*What is your race? Choose all that apply*" with response options including 1) White, 2) Black or African American, 3) American Indian or Alaska Native, 4) Asian Indian, 5) Chinese, 6) Filipino, 7) Japanese, 8) Korean, 9) Vietnamese, 10) Other Asian, 11) Native Hawaiian, 12) Guamanian or Chamorro, 13) Samoan, and 14) Other Pacific Islander. These categories were then combined and reported by the PATH Study using the following 4 categories: 1) White alone, 2) Black alone, 3)Asian race alone (defined as including Asian Indian, Chinese, Filipino, Japanese, Korean, Vietnamese, or Other Asian and could include multiple Asian categories), 4)Other race, multiracial (defined as including Native Hawaiian, Guamanian or Chamorro, Samoan, and Other Pacific Islander or a combination of >1 race categories).

Ethnicity was assessed via the question "*Are you Hispanic, (Latino, Latina, Latino or Latina), or of Spanish origin? Choose all that apply*" with response options including 1) No, not of Hispanic, (Latino, Latina, Latino or Latina), or Spanish origin, 2) Yes, Mexican, Mexican American, (Chicano, Chicana, Chicano or Chicana), 3) Yes, Puerto Rican, 4) Yes, Cuban, or 5) Yes, another Hispanic, (Latino, Latina, Latino or Latina), or Spanish origin. These categories were then combined and reported by the PATH Study using the following two categories: 1) Hispanic (yes response to options 2-5), and 2) Not Hispanic (yes to response 1).

Race and ethnicity, along with sexual orientation, were assessed to assist in developing population-based weights and specific sub-group analyses on tobacco use. When race and ethnicity were not available, imputation methods when used to determine race and ethnicity. More details regarding imputation methods can be found in the PATH User Study Guide at: https://www.icpsr.umich.edu/files/NAHDAP/pathstudy/ug36231-all_REST.pdf.

The use of Wave 7 cross-sectional weights allowed for response rates to be used to estimate the U.S. civilian, non-institutionalized population rates of use. We used within group weighting for product substitution, NRT use, and prescription medications to better represent use of specific strategies amongst this population.

Results reporting for our study adhered to STROBE guidelines.

© 2025 Williams BS et al. *JAMA Network Open*.
