# Supplementary material for: Vaping Cessation Methods Used by Young Adults
Source: JAMA Netw Open. 2025 May 29;8(5):e2512803. doi: 10.1001/jamanetworkopen.2025.12803 (PMC12123465; doi:10.1001/jamanetworkopen.2025.12803)
Supplement: Supplement 2. — Data Sharing Statement [file jamanetwopen-e2512803-s002.pdf]

Data Sharing Statement

Williams. Vaping Cessation Methods Used by Young Adults. *JAMA Netw Open*. Published May 29, 2025. doi:10.1001/jamanetworkopen.2025.12803

Data

Data available: No

Additional Information

Explanation for why data not available: PATH Study data is publicly available in the PATH Study database at https://www.icpsr.umich.edu/web/NAHDAP/series/606.
